# Understanding host–pathogen interaction: paving the path for individualized anti-infective therapy

**DOI:** 10.1007/s00430-024-00787-y

**Published:** 2024-04-10

**Authors:** Isabelle Bekeredjian-Ding

**Affiliations:** 1 Institute for Medical Microbiology and Hospital Hygiene, Philipps University Marburg, Marburg, Germany; 2 DZIF (German Center for Infectious Disease Research) Partner Site Giessen-Marburg, Marburg, Germany

In the COVID-19 pandemic, we were challenged by the spread of SARS-CoV2, a virus not confined to borders nor easy to contain. The virus took its toll in vulnerable populations until—for the first time in the history of mankind—the pathogen was contained by deployment of vaccines developed in breathtaking speed. Retrospectively, the pandemic turned into a unique opportunity for researchers and developers to exploit their knowledge and technologies to combat the virus. Robert Koch, the famous German microbiologist, who founded this journal as the “*Zeitschrift für Hygiene und Infectionskrankheiten*” in 1886 with Carl Flügge, would have been amazed! In his days, he spent significant time traveling abroad to characterize infection and contagion by pathogens such as *M. tuberculosis*, *V. cholera*, *Y. pestis*, Malaria-causing plasmodia and *Trypanosoma cruzei*, and developed and tested new therapeutic and preventive approaches. Needless to say, that most of these pathogens remain meaningful global health threats due to our still incomplete understanding of pathogenesis and immune defense and their recently increasing mobility across the globe.

Today, globalization, urbanization and climate change have altered the dynamics of infectious disease spread. They facilitate transmission of infectious pathogens by increasing the frequency of direct contacts among humans, with animals and over large distances, and promote favorable growth conditions for microbes and the reproduction of vectors that harbor infectious pathogens. Robert Koch and Carl Flügge were well aware that transmission of an infectious agent and manifestation of infection strongly depends on host susceptibility and virulence traits of the pathogen. Well in line with their investigations, the scope of this journal was, thus, traditionally centered on host–pathogen interaction. To date, sophisticated tools for genetic, molecular and functional analysis of infecting microbes are available and, in parallel, we have the means for performing a thorough analysis of host genetics and immune status capturing the molecular correlates of susceptibility of individuals to infection and carriage. However, the available methodology is not yet comprehensively exploited.

In spite of this, there is an emerging demand to extend analyses to enable prediction of efficacy of therapeutics and vaccines in the individual. Awareness that there is no “one size fits all” has surged a new paradigm in therapeutic decision making in many fields of medicine, and also applies to vaccines and anti-infectives. However, implementation of individualized approaches to diagnostics and therapy requires more research to define the relevant genetic and immunological variation within a population and the correlation of these features with individual therapeutic or vaccine responses. This can range from ex vivo experimental and in silico models to clinical trials and real-world evidence studies that report and provide proof on predictive factors that need to be diagnosed before choosing from a portfolio of anti-infective vaccines or therapeutic options and becomes even more relevant when thinking of the evolving field of host-directed therapies.

Notably, the need for individualized diagnostics and therapy is not new but the increasing demand is an important indicator of a profound perceptional change in the way we view and perform medicine. For example, we are experiencing a change when the priority in an immunization campaign is no longer or not only evaluated through the lens of achieving herd immunity but gains a new dimension through a spotlight on prevention of infection (and ideally colonization) in the individual. This translates into increasingly sophisticated vaccine recommendations on subpopulation level and opens the path for targeted new vaccine and medicines developments.

This individual-centered point of departure can be viewed as a direct consequence of today’s high-tech medicine. It has brought about new complexity through prolonging lives and identifying and enabling the diagnosis of a broad spectrum of risk factors and immunocompromised conditions. Awareness of the risks and the associated uncertainty of therapeutic outcome justifies the investment into patient-centered stratification of diagnostics and therapeutics. While many infectious disease specialists will argue that concepts for screening for multidrug resistant pathogens, antibiotic stewardship and choice of antivirals already provide this individualistic approach, my personal point of view is that this trend is more fundamental. It might reflect a change of mindset on a societal level that can be subsumed as the longing for facts and understanding of a self-determined and literate patient. This patient asks for a root cause analysis (causative pathogen and susceptibility profile) and, what is more, a warranty for protection (probability regarding the individual treatment choice and outcome and alternatives). However, we are not yet ready for this. The field requires more research and exploration to address the multitude of factors that influence host–pathogen interactions and drive clinical outcomes.

**Fig. 1:**
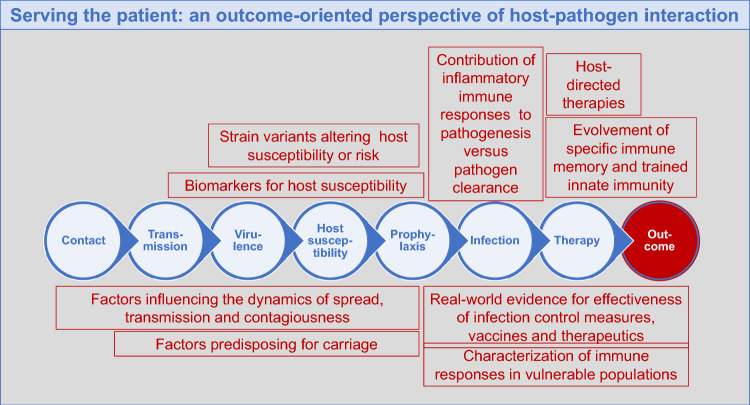
Overview on the life span of an infection and relevant topics for MMIM

How can *Medical Microbiology and Immunology* contribute to the field? Science is continuously evolving, we need to keep track and move on. As the new editor-in-chief, I will propagate MMIM as a forward-looking journal that captures trends and advances in the understanding of the host–pathogen interaction ensuring a clinical outcome-oriented perspective and showcasing the possibilities to exploit new data and findings for the benefit of the patient. The outcome-oriented approach and the patient context influence the relevancy of findings and the understanding of the reader. Examples for relevant topics in the life cycle of infection are summarized in Fig. 1.

How can *MMIM* ensure impact? The goal is to make a difference by actively seeking and focusing on articles that address variability of host and pathogen traits and link these to patient outcome and therapy.

Finally, I would like to thank all the authors, reviewers and editors that have contributed the MMIM in the last decades. A detailed understanding of the host–pathogen interaction will remain the only basis for new approaches to treatment and prevention of infectious disease. I would, therefore, want to encourage all of you to actively seek innovative solutions to infectious disease problems and foster a critical discussion about data, observed trends and prospective threats.

